# A wearable heart rate measurement device for children with autism spectrum disorder

**DOI:** 10.1038/s41598-020-75768-1

**Published:** 2020-10-29

**Authors:** Francesca Fioriello, Andrea Maugeri, Livio D’Alvia, Erika Pittella, Emanuele Piuzzi, Emanuele Rizzuto, Zaccaria Del Prete, Filippo Manti, Carla Sogos

**Affiliations:** 1 Department of Human Neuroscience, Sapienza University of Rome, 00185 Rome, Italy; 2 Department of Mechanical and Aerospace Engineering, Sapienza University of Rome, 00184 Rome, Italy; 3 Department of Legal and Economic Sciences, Pegaso University, 00184 Rome, Italy; 4 Department of Information Engineering, Electronics and Telecommunications, Sapienza University of Rome, 00184 Rome, Italy

**Keywords:** Neuroscience, Psychology

## Abstract

Autism spectrum disorder (ASD) is a neurodevelopmental condition characterized by early impairment in social and communication domains and autonomic nervous system unbalance. This study evaluated heart rate (HR) as a possible indicator of stress response in children with ASD as compared to children with language disorder (LD). Twenty-four patients [mean age = 42.62 months; SD = 8.14 months,12 with ASD (10 M/2F) and 12 with LD (8 M/4F)] underwent clinical [Leiter International Performance Scale-Revised, Autism Diagnostic Observation Schedule, second edition (ADOS-2)] and physiological evaluation (HR monitoring) during five interactive activities, while wearing an HR measurement device. IQ (ASD:IQ = 103.33 ± 12.85 vs. LD:IQ = 111.00 ± 8.88, *p* = 0.103) and fluid reasoning on the Leiter-R Scale were within the normal range in all subjects. Increased HR during the third activity (ADOS-2 bubble play) significantly correlated with autistic symptoms (*r* = 0.415; *p* = 0.044), while correlations between ADOS-2 total score and HR during the first activity (ADOS-2 free play; r = 0.368; *p* = 0.077), second activity (Leiter-R figure ground subscale; r = 0.373, *p* = 0.073), and fifth activity (ADOS-2 anticipation of a routine with objects; r = 0.368; *p* = 0.076) did not quite reach statistical significance. Applying a linear regression model, we found that the ADOS-2 total score significantly influenced HR variations (*p* = 0.023). HR monitoring may provide a better understanding of the stress-provoking situations for children with ASD. Furthermore, it could help clinicians detect the impact of the stressful condition on the autistic core and adress treatment strategy.

## Introduction

Autism spectrum disorder (ASD) is a neurodevelopmental disorder characterized by social and communication impairment and restricted and repetitive patterns of behavior and interests. ASD symptoms can be detected between 12 and 24 months of age in some children^1^. ASD prevalence estimates varied among sites, from 1 to 54 children aged 8 years. Males were four times more likely than females to be diagnosed with ASD^2^.

Children with ASD often have difficulties understanding and recognizing their emotions, therefore making it difficult to infer when they are experiencing stress^3^. However, non-invasive wearable devices have recently introduced the possibility to analyze and monitor symptoms of stress in children with neurodevelopmental disorders.

In recent years, wearable devices have played a crucial role in many different applications, including occupational accident prevention, fitness, and medical care, thanks to the ability to combine many low-cost sensors in the same network^4–6^. In the healthcare field, user-friendly wearable devices allow vital signs to be measured and monitored with different granularity levels. They provide significant benefits, including real-time diagnostic screening in conditions and environments in which traditional instrumentation and tools cannot be applied. Wearable devices permit physiological (i.e., temperature, oxygen saturation, pulse, blood pressure), movement and gait, and cardiac and respiratory parameters to be evaluated^7–9^.

Several theoretical models have linked autistic symptoms to possible autonomic nervous system (ANS) dysfunction involved in response to stress and anxiety^10,11^. The ANS is a complex system of feedback pathways that links cardiac activity with central nervous system processes. The ANS may therefore play a key role in regulating social functioning in children with ASD and may also influence cognitive, affective, and behavioral responses in these subjects^12–15^.

Several approaches can be used to assess ANS activity through heart rate (HR) and heart rate variability (HRV) measures^16–18^. In typically developing toddlers and preschool-age children, HR and HRV relate to emotional reactivity and social skills^19,20^. Several studies have demonstrated the role of HRV as an established measure of regulated emotional response in typically developing populations^21–23^. These physiological signals can be used to identify stress response and its relationship to potentially challenging behaviors.

Hoch et al.^24^ explored the link between arousal and behavioral in ASD children and showed that activity choices were sequentially dependent on the preceding level of HR.

Nuske et al.^25^ showed less concordance between internal physiological arousal and external emotional communication in children with ASD. Therefore, it may be difficult for caregivers to recognize stressful events and manage and minimize challenging behaviors, thus emphasizing the importance of understanding triggers.

HRV in response to cognitive or psychosocial stress has been explored very little in preschool-age children with ASD^26^. While some studies have reported no significant differences in ANS activity in children with ASD as compared to typically developing children, others have shown blunted autonomic responses to visual and auditory social stimuli^27^. Lastly, increased HR during REM sleep has been observed in ASD subjects as compared with matched healthy controls^28^.

We hypothesized that increased HR was associated with higher stress levels in preschoolers with ASD.

In order to gain a better understanding of the ASD emotional state and to plan specific treatment, it is necessary to determine whether ASD patients experience more anxiety when they are involved in interactive situations or when they are isolated or making stereotypies. Thus, this study aimed to evaluate the HR as observed during different interactive activities as a possible indicator of stress response in children affected by ASD as compared to children with language disorder (LD). We also explored whether this association varied according to gender, age, or cognitive ability. We applied a small wearable thoracic belt, suitable for children, that was sensorized with three electrocardiograph and a piezoelectric sensor in order to monitor cardiorespiratory activity in children with ASD and LD. The monitoring system included a microcontroller with a wireless interface able to transmit the acquired parameters to a personal computer for post-processing analysis.

## Materials and methods

### Participants

Fifty-four subjects who met the criteria for ASD or LD diagnosis were recruited from patients with a neurodevelopmental disorder who were diagnosed and received follow-up at the Department of Human Neuroscience of Sapienza University of Rome. Inclusion criteria were: (a) age range 30–72 months; (b) IQ ≥ 85; and c) a diagnosis of ASD or LD without verbal comprehension deficit. Exclusion criteria were: (a) the presence of neurological defects or neurological deterioration; (b) signs of cardiovascular disease or a history of other illnesses (visual impairment or hearing loss); (c) the use of psychotropic medications, including melatonin (for at least 72 h before testing); or (d) genetic conditions of known etiology (such as fragile X syndrome, or chromosomal abnormalities). The study was approved by the Ethics Committee of Sapienza University of Rome and performed in compliance with the Declaration of Helsinki (2000), and it was performed under these guidelines and regulations. Written informed consent was obtained from the subjects’ parents.

### Study design

#### Clinical assessment

All children underwent a full clinical evaluation, including medical history, clinical observations, and neuropsychiatric assessment. Diagnoses were made according to Diagnostic and Statistical Manual of Mental Disorders, fifth-edition (DSM-5) criteria.

The Leiter International Performance Scale-Revised (Leiter-R) was used^28^ to evaluate the cognitive functioning. This scale is a non-verbal measure of intelligence consisting of 20 sub-tests divided into a Visualization and Reasoning Battery and an Attention and Memory Battery (for individuals aged between 2 and 20 years, 11 months). In this study, only the Leiter-R Visualization and Reasoning Battery was used.

The Autism Diagnostic Observation Schedule-second edition (ADOS-2) was used to assess the presence of autism features^29^. The ADOS-2 is a direct semi-structured assessment composed of five modules (depending on the age and language abilities of the subject). For all modules, calibrated severity scores could be derived from the total score as well as from the social affect and restricted and repetitive behaviors domains.

#### Hardware and software interface

**Figure 1:**
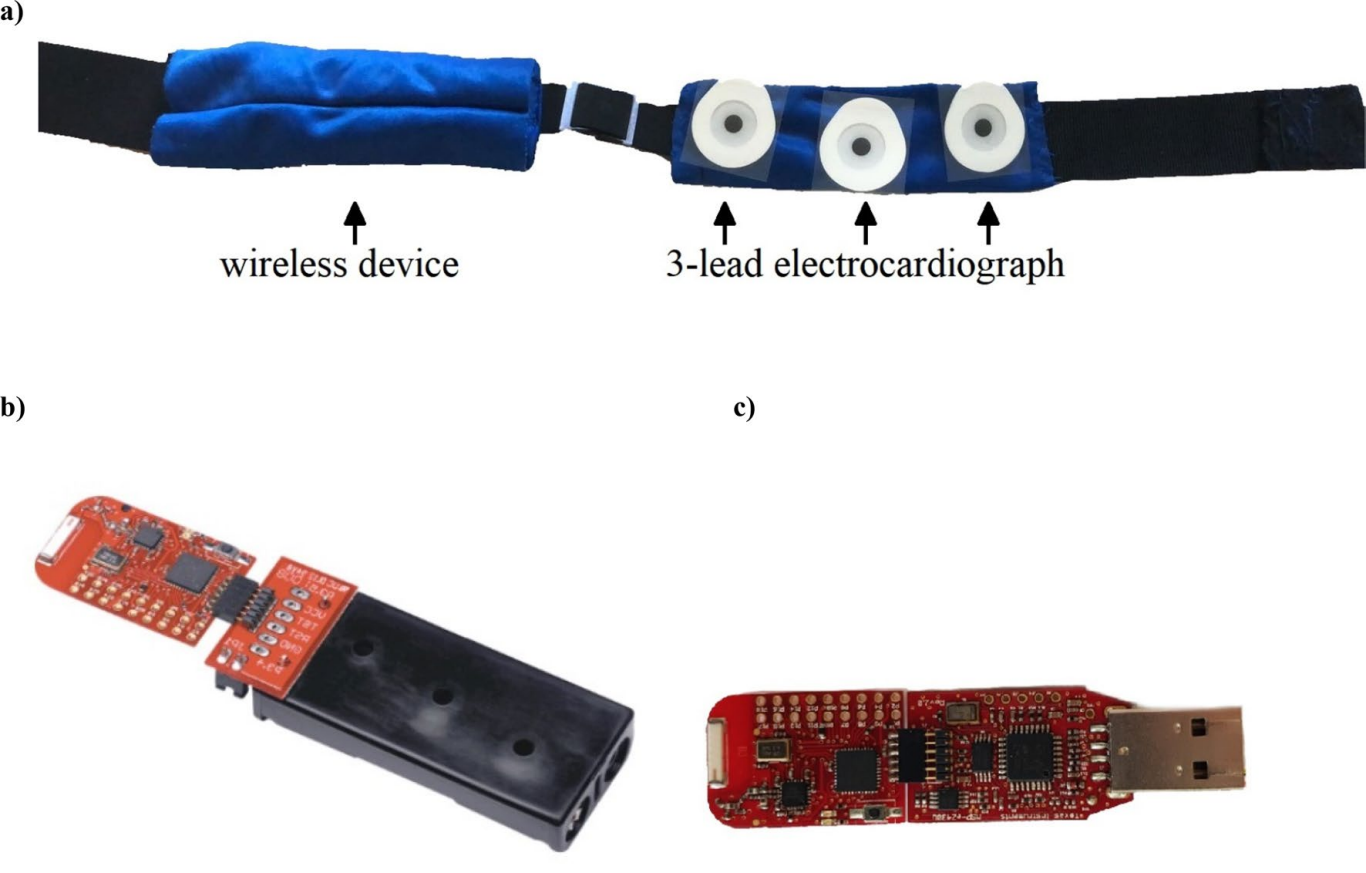
Heart rate device. (**a**) thoracic belt with highlighted the 3-lead electrocardiograph; (**b**) the wireless device embedded in the belt; (**c**) the USB transceiver.

The wearable sensor device used was a low cost (~ $50) system that integrated a three-lead electrocardiograph (ECG) sensor for cardiac activity monitoring (Fig. 1). The system was characterized by an *ad-hoc* prototype circuit board in which the signals from the three-way ECG were firstly amplified by an instrumentation amplifier (AD8220) to eliminate the common-mode component. Due to its common-mode rejection ratio. A second stage was based on an OP2177, precision amplifier that further compensated the common-mode caused by body disturbances and the power supply circuit. Thirdly, an operational amplifier (LMV824N) was connected to filter (high-pass filter with a cut-off frequency of 0.034 Hz) and offset baseline and low-frequency motion artifacts. Finally, the signal was sampled at 100 Hz by the embedded microcontroller (MSP430). The present circuit provided a connector to investigate breathing activity for future applications.

Regarding the wireless sensors network, the belt embedded the communication target board from the eZ430-RF2500 development tool, which integrated the MSP430, a low-consumption, low-cost, and small-size microcontroller, and the CC2500, a low-power 2.4-GHz wireless transceiver.

The second USB target was connected to a personal computer where data were acquired in real time, plotted, stored, and analyzed through a virtual instrument developed in the LabVIEW environment. Radio transmission between the two boards was guaranteed due to a 2.4 GHz antenna integrated into the printed circuit. In the LabVIEW virtual instrument, the heart signal (beats per minute) was filtered and processed and then presented on the front panel through a proper graph. Simultaneously, processed data were stored for postprocessing analysis using MATLAB and SPSS.

#### Experimental protocol

The children were monitored 6 months after the diagnostic assessment, using a wearable belt system during five activities specifically designed to evaluate competencies related to inter-subjectivity, joint attention, shared enjoyment, and communication with the operator. The experimental protocol started with the ADOS-2 free play activity (activity 1) in order to allow the child to settle in and become familiar with the operator. The second and fourth activities (figure ground and form completion subtests) were part of the Leiter-R scale and were chosen as the models of structured desk activities.

The third and fifth activities, namely ADOS-2 bubble play and anticipation of a routine with objects, were chosen to evaluate the HR frequency in joint-attention and shared enjoyment in interactions.

All activities were recorded and the variation in the HR frequency was subsequently analyzed in relation to the specific activity carried out.

Evaluating HR variations allowed the response to different types of stressful events to be identified and compared between children with ASD and those with LD. In particular, HR may be useful to verify differential stress responses during tasks with more significant social interaction, which is compromised in children with ASD. For this purpose, tests were used that provided different degrees of interactions between the child and operator. The entire study protocol took approximately 20 min, with at least 4 min for each activity.

### Data analysis

ECG signal was evaluated and analysed in the offline MATLAB environment. Firstly, the proposed considered significant ECG QRS peaks (QRSp), which were selected based on: (i) peak magnitude, and (ii) the distance between two significatant peaks.

Second, the instant heart rate (IHR) was assessed via the QRSp assessment. We considered the average heart rate fM [beats per minute (bpm)] and its variation in terms of standard deviation σ (bpm) for each activity. Furthermore, a moving average filter with a span of 10 s was applied to minimize errors produced by uncontrolled belt movements or subjects upper limb movements.

All statistical analyses were conducted using IBM SPSS Statistics version 23.0 (SPSS Inc., Chicago, Illinois, USA). A probability value of < 0.05 was chosen as the level of significance for all tests. Normality was assessed with the Kolmogorov–Smirnov test. Comparisons were made using the independent sample t-test for continuous variables and the Fischer’s exact test for categorical variables. Spearman's correlation coefficient was used to analyze correlations. The effect of brief IQ, fluid reasoning, and ADOS-2 total scores on HR activity was evaluated by linear regression analysis.

## Results

Fifty-four participants were recruited for the study. Thirty children (13 with ASD and 17 with LD) dropped out due to poor compliance during the protocol administration (missing test or incomplete evaluation).

The final sample consisted of 24 subjects [mean age = 42.62 months; SD = 8.14 months, 12 with ASD (10 M/2F) and 12 with LD (8 M/4F)]. There were no significant gender differences between groups (Fisher’seExact test, *p* = 0.640).

Mean IQ (ASD: IQ = 103.33 ± 12.85 vs. LD: IQ = 111.00 ± 8.88, *p* = 0.103) and fluid reasoning (FR) scores were within the normal range in all subjects, and we observed a strong trend towards a statistically significant difference in the FR scores between the two groups (ASD: FR = 91.92 ± 11.37 vs. LD: FR = 111.33 ± 11.63, *p* = 0.057).

**Figure 2:**
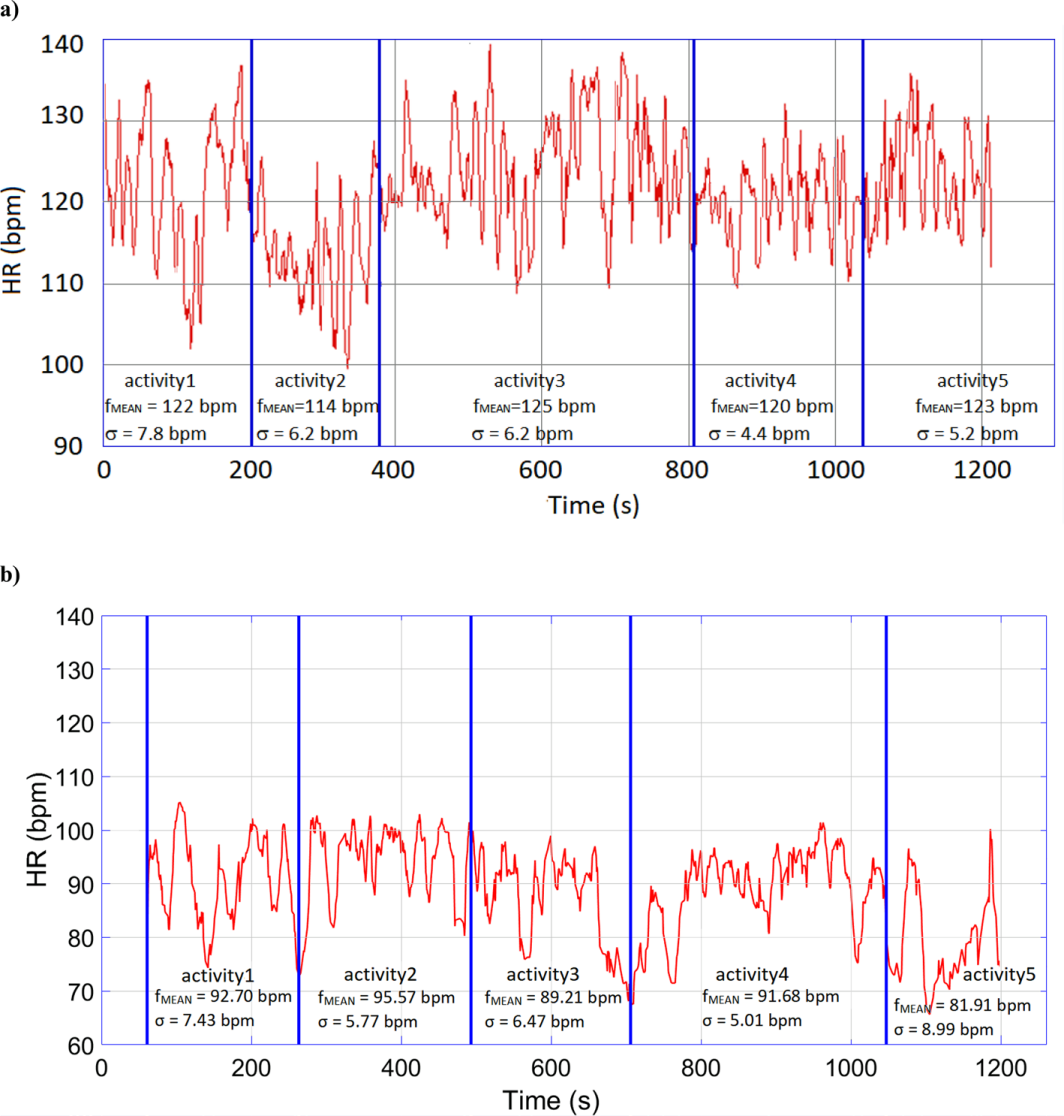
Heart rate variations during the proposed activities in a subject with ASD (**a**) and in a subject with LD (**b**). *HR* heart rate, *bpm* beats per minute, *ASD* autism spectrum disorder, *LD* language disorder.

Average HR during the entire protocol and for each single activity was significantly higher in patients with ASD as compared to those with LD (Table 1, Fig. 2).

**Table 1:** T-test comparison between ASD and LD groups.

	ASD group (M ± SD)	LD group (M ± SD)	t	df	*p*
Age (months)	43.66 ± 8.65	41.58 ± 7.84	0.618	22	0.543
Brief IQ (Leiter-R)	103.33 ± 12.85	111.00 ± 8.88	− 1.700	22	0.103
Fluid Reasoning (Leiter-R)	91.92 ± 11.37	101.33 ± 11.63	− 2.005	22	0.057
Figure Ground (SS) (Leiter-R subtest)	13.00 ± 2.69	12.67 ± 2.29	0.816	22	0.423
Form Completion (SS) (Leiter-R subtest)	11.75 ± 2.34	13.92 ± 2.06	− 2.405	22	0.025*
ADOS-2 total score (severity score)	5.58 ± 1.38	0.33 ± 0.49	12.421	22	0.000**
1st activity (HR) Free play activity (ADOS-2)	113.92 ± 9.99	103.95 ± 9.64	2.487	22	0.025*
2nd activity (HR) Figure ground (Leiter-R subtest)	110.91 ± 9.48	101.99 ± 9.67	2.279	22	0.033*
3rd activity (HR) Bubble play activity (ADOS-2)	114.22 ± 10.89	102.27 ± 13.56	2.379	22	0.026*
4th activity (HR) Form completion (Leiter-R subtest)	112.60 ± 9.63	104.56 ± 10.88	1.916	22	0.068
5th activity (HR) Anticipation of a routine with objects activity (ADOS-2)	114.61 ± 11.02	101.73 ± 12.07	2.731	22	0.012*
Total activities (HR)	113.25 ± 9.69	102.75 ± 10.22	2.581	22	0.017*

*ADOS-2* autism diagnostic observation schedule, *ASD* autism spectrum disorder, *LD* language disorder, *HR* heart rate, *Leiter-R* Leiter international performance scale-revised, *SS* scaled scores.

*p < 0.05; **p < 0.01.

Furthermore, we observed that children with ASD more frequently approached the upper limit of the normal HR range for age (95–140 bpm for 3–5 years of age), with 4 patients over 120 bpm on average in the ASD group vs. 0 in the LD group.

For the entire protocol and for each single activity, no significant differences were found in HR standard deviation (HRsd) between the two groups.

Increased HR during the third activity (ADOS-2 bubble play) significantly correlated with autistic symptoms (*r* = 0.415; *p* = 0.044), while correlations between ADOS-2 total score and HR in the first activity (ADOS-2 free play; r = 0.368; *p* = 0.077), second activity (Leiter-R figure ground subscale; r = 0.373, *p* = 0.073), and fifth activity (ADOS-2 anticipation of a routine with objects; r = 0.368; *p* = 0.076) did not quite reach statistical significance (Fig. 2).

Finally, linear regression analysis was performed. A stepwise method that included total HR during the activities as well as brief IQ, FR, and ADOS-2 total scores at diagnosis as independent variables was applied. We found a significant effect of ADOS-2 total score on HR during the activities (*p* = 0.023), and the percentage of variance explained was higher (21%, R^2^ = 0.212). The model excluded brief IQ and FR variables.

## Discussion

We monitored the HR in children with ASD and LD during five interactive activities involving stress responsivity and the joint attention. This pilot study found that the HR of children with ASD was significantly higher than the HR of children with LD during interactive activities. In particular, HR differences were more relevant when the proposed activity involved emotional involvement or expected shared enjoynment (as in the bubble play or anticipation of a routine with objects activities).

The increased HR that we found in ASD children seems to confirm that they experience stress during all interactive activities, with a low capability to modulate the emotional response process. HR was more elevated and less modulated in children with ASD as compared to children with LD.

Furthermore, we observed a link between autism severity scores (ADOS-2) and HR during the interactive activities. The more severe the autistic symptoms; the higher the HR during the study activities.

These results support the hypothesis that both children with ASD and LD showed emotional activation during interactive activities. Nevertheless, interactive activities seemed to be stressful for children with ASD.

Few previous investigations have measured physiological parameters in very young children with ASD^30^, and most of these studies have focused on the emotional recognition abilities of children with ASD, rather than on the management of their emotions^31^.

A cross-sectional study by Corona et al.^32^ demonstrated that mentally retarded children aged 3–5-years showed an HR decrease during the distress condition, but the HR of the children with ASD did not change across the same conditions. In another study, Watson et al.^33^ found an increased HR in children with ASD as compared to children with typical development, during non-social and child-directed speech stimuli. These results were attributed to an overactive sympathetic system, an underactive parasympathetic system, or both.

Evidence from our study suggests an adverse effect of autistic symptomatology on outcome, especially in terms of joint attention and social cognition. We hypothesized that higher brain vulnerability to stress conditions during the early critical period of brain maturation should determines a stable impairment of cognitive and emotional functions, as argued in the work of Mundy and Jarrod^34^.

Joint attention is seen as an executive social function of the frontal-parietal neural network, which is essential for the functional gain in the efficiency of social interactions^34^.

Social cognition ability is necessary in the development and use of symbolic thinking throughout the life span^35^.

Notably, the two clinical groups showed different focus during the activities. While children with ASD were more focused on the object rather than on the relationship with the operator, children with LD showed an evident and prevalent interest in the operator. These findings confirm the presence of a severe impairment in initiating and sustaining joint attention in children with ASD and support the hypothesis that interactive activities are stressful situations for these children. This evidence might suggest that all the everyday experiences that involve interacting with adults or peers are potentially stressful for children with ASD.

Furthermore, Nuske et al.^25^ reported that HR may predict challenging behavior in children with ASD and support difficulties in communication. Data analysis showed different results and HR profiles than we expected, with a similar HRsd obtained in all activities, in the clinical sample, suggesting a poor adaptation and a high level of stress in children with ASD. These findings seem to be confirmed when compared with the free play activity highlighting frequent stress demonstrations already evident during the sensory belt wearing phase, with no difference between the two groups. However, the persistent and significantly higher mean HR value, in addition to the slightly (although not significant) lower standard deviation in the ASD group, also corresponded with qualitative differences (mostly regarding social interaction), suggesting higher stress in children with ASD.

This finding is in line with the study of Kushki et al.^36^ which reported increased sympathetic activity in children with ASD. In contrast, children with a typical development exhibited a decreased sympathetic influence during social interaction with parents^37,38^.

## Conclusion

The present study describes the use of an HR measurement device as a non-invasive and painless objective measure of stress in preschoolers with ASD. This device seems to be particularly useful since most subjects with ASD cannot recognize and express external and internal symptoms of stress. Our preliminary findings also introduce the possibility of using HR variations as possible indicators of stress response during clinical evaluation and in daily life in children with ASD. These results could also be promising targets for stress prevention and tailored treatment.

Furthermore, HR monitoring appears to be a reliable method to test ANS functionality in children with other neurodevelopmental disorders and psychiatric conditions (such as anxiety or depressive disorders).

The main limitation of our study is the small sample size. Further data collection is needed to confirm our preliminary results, in order to characterize physiological patterns likely to be linked to different behaviors and emotional states and to monitor the outcome.
